# Functions, Roles, and Biological Processes of Ferroptosis-Related Genes in Renal Cancer: A Pan-Renal Cancer Analysis

**DOI:** 10.3389/fonc.2021.697697

**Published:** 2022-03-11

**Authors:** Linbao Chen, Chao Wang, Yuning Wang, Tianyu Hong, Guangwen Zhang, Xingang Cui

**Affiliations:** ^1^ Department of Urinary Surgery, The Second Affiliated Hospital of Ningxia Medical University (The First People’s Hospital of Yinchuan), Yinchuan, China; ^2^ Ningxia Medical University, Yinchuan, China; ^3^ Department of Urinary Surgery, Postgraduate Training Base in Shanghai Gongli Hospital, Ningxia Medical University, Yinchuan, China; ^4^ Department of Urinary Surgery, Gongli Hospital, Second Military Medical University (Naval Medical University), Shanghai, China; ^5^ Department of Urology, The Affiliated Changzhou No. 2 People’s Hospital of Nanjing Medical University, Changzhou, China; ^6^ Department of Urinary Surgery, Xinhua Hospital Affiliated To Shanghai Jiaotong University School of Medicine, Shanghai, China

**Keywords:** ferroptosis, kidney cancer, weighted gene co-expression network analysis, pan-renal cancer analysis, bioinformatics analysis

## Abstract

Ferroptosis is a cell death process discovered in recent years, highly related to cancer, acute kidney injury, and other diseases. In this study, a pan-renal cancer analysis of ferroptosis-associated genes in renal cancer was performed to construct a multigene joint signature for predicting prognosis in renal cancer patients. First, gene expression profiles were downloaded from the TCGA and GTEx databases to search for genes significantly associated with renal cancer prognosis through differential gene expression analysis, weighted gene co-expression network analysis (WGCNA), and survival analysis. Thereafter, the gene-set enrichment analysis (GSEA) was used to identify the biological processes in which ferroptosis-associated genes might be involved. Weighted gene co-expression network analysis resulted in 4,434 differentially expressed genes (DEGs) and 42 co-expression modules, among which ferroptosis-related genes were distributed in 11 gene modules. The survival analysis screening resulted in three DEGs associated with renal cancer prognosis, namely SLC7A11, HMOX1, and MT1G. Specifically, SLC7A11 and HMOX1 were upregulated in renal cancer tissues, while MT1G was downregulated. Receiver operating characteristic (ROC) curves, combined with Kaplan–Meier and Cox regression analysis, revealed that high expression of SLC7A11 was a prognostic risk factor for four different renal cancers, that low expression of HMOX1 was a poor prognostic marker for patients, and that increased expression of MT1G increased the prognostic risk for three additional classes of renal cancer patients, except for renal papillary cell carcinoma. The GSEA results showed that the ferroptosis-related genes from these screens were mainly associated with signaling pathways related to tumor progression and tumor immunity. This study provides potential biological markers for prognosis prediction in renal cancer patients with different subtypes, and these results imply that ferroptosis is highly associated with renal carcinogenesis progression.

## Introduction

Renal cancer is a large heterogeneous group of cancers derived from renal tubular cells, and, as the seventh most common malignancy worldwide, the incidence is still increasing (1). Kidney cancer comprises dozens of distinct molecular and histopathological subtypes, among which renal clear cell carcinoma (KIRC) accounts for approximately 75% of all kidney cancers; renal papillary cell carcinoma (KIRP) and renal chromophobe carcinoma (KICH) account for 15% and 5%, respectively (2). There are large differences between the clinical outcomes of different renal cancer patients’ subtypes, which reflect the complexity of cancer biology and the heterogeneity of the effects of oncology drugs (3, 4). However, the typical clinical symptoms of renal cancer are not specific, and the onset of renal cancer is insidious, leading to the diagnosis and treatment of renal cancer patients often not being timely; as a result, most patients are diagnosed combined with distant metastasis or advanced tumor stage, and eventually die of the disease (3). Further, even with radical surgery targeting the lesion, there will be a relapse in around 40% of patients (5). Therefore, seeking the possible pathogenesis of kidney cancer and dissecting its key functional molecules could provide new perspectives for prognostic models in patients with kidney cancer. Notably, because of the unique anatomical localization of the adrenal gland, adrenocortical carcinoma (ACC), as an aggressive growing malignant tumor, often metastasizes into the kidney. Clinical treatment often combines it with the adjacent affected kidney and resection (6, 7), so this study combined ACC with other renal cancer subtypes for analysis.

Ferroptosis is an iron-dependent novel programmed cell death mechanism. The distinction between ferroptosis and autophagy is that cells undergoing ferroptosis have increased mitochondrial membrane density and decreased mitochondrial cristae (8). Currently, it has been found that ferroptosis plays a crucial regulatory role in several diseases, such as cancer, acute kidney injury, and neurological disorders, and blocking or activating the cellular ferroptosis pathway could provide therapeutic strategies with great potential for these diseases (9, 10). Studies have pointed out that KIRC and ACC cells rely heavily on glutathione (GSH) and glutathione peroxidase (GPx) to decrease the lipid peroxidation level of tumor cells, which illustrates that tumor growth can be inhibited by inducing cell ferroptosis (11, 12). Gene signatures associated with ferroptosis have been highly correlated with the clinical and pathological features of gliomas. They can serve as reliable indicators for the prognostic evaluation of glioma patients (13). Ferroptosis-related genes such as GPX4 (14), NFE2L2 (15), and NOX1 (16) have been found to play a crucial role in tumorigenesis and progression, and these molecules are potentially essential players for cancer treatment and prognosis evaluation.

Given that the current expression pattern of pro-ferroptosis genes and their links in renal cancer is not clear, to revealed ferroptosis-related genes in renal cancer and assess their possible predictive value, we analyzed the distribution of ferroptosis-related genes in each gene module by screening differentially expressed genes (DEGs) from TCGA renal cancer expression profiles, and then constructed co-expressed gene modules. The DEGs of ferroptosis associated with prognosis were screened by univariate Cox regression analysis and the Kaplan–Meier test. Finally, a pan-renal cancer analysis was combined to evaluate the prognostic significance of each gene signature in different renal cancer subtypes.

## Method

### Data Collection

Gene expression data and related clinical information were obtained from The Cancer Genome Atlas (TCGA) database and the Genotype-Tissue Expression (GTEx) database. ACC, KICH, KIRC, and KIRP expression data from TCGA were merged and subsequently processed to remove batch effects, and the data were matched to the corresponding clinical samples. Cases with duplications, deletions, and missing clinical outcomes were excluded.

### Differential Gene Expression Analysis

The gene count values of the samples were differentially analyzed using the R software DEseq2, and the DEGs were obtained by filtering with |log2 (FC) | > 2, P < 0.05. The expression pattern clustering heat map and volcano plots were drawn using the R software, ggplot.

### Weighted Gene Co-Expression Network Analysis

Gene modules co-expressed with genes associated with sample characteristics in renal cancer samples were identified using the R software, WGCNA. The expression matrix of the samples was first log-transformed, sample clustering was used to identify outlier outliers in the samples, a soft threshold was calculated, and a matrix was built. Hierarchical clustering and dynamic clipping were used to detect modules with a minimum module gene count of 30 and a cut height of 0.3. Finally, correlation higher than 0.75 were merged and the others cropped.

### Survival Analysis

Survival analysis was performed using the R software called survival. Hazard ratios (HR) and 95% confidence intervals (CI) were first calculated by verifying the association between expression levels of 60 ferroptosis-related genes and overall survival (OS) of renal cancer patients using the univariate Cox regression analysis. The Kaplan–Meier test was then performed to analyze the difference between the survival of patients with high and low expression of genes.

### The Gene-Set Enrichment Analysis

Samples were divided into two groups with high and low expression according to the median gene expression, and the GSEA was used to seek the effect of gene expression on tumors. Filtering criteria were FDR < 0.25, P < 0.05.

## Result

### Screening of DEGs in Renal Cancer

**Figure 1:**
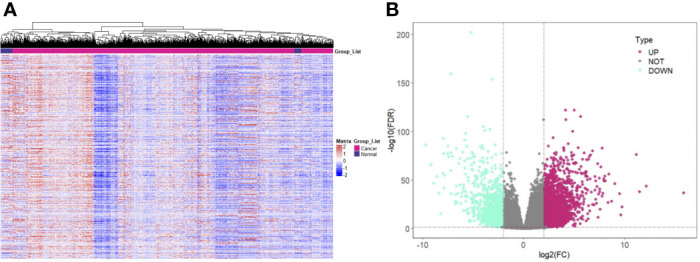
Screening of DEGs in Renal Cancer. **(A)** Expression pattern clustering heat map; **(B)** Volcano plot of DEGs. Note: DEGs: Differentially expressed genes; Teal for downregulated DEGs and purple-red for upregulated DEGs.

After preprocessing the data in TCGA, 964 samples were obtained, including 907 tumor samples and 57 normal samples. The heat map was plotted with the expression quantity of genes in individual samples (**Figure 1A**). The resulting 3,384 upregulated DEGs and 1,050 downregulated DEGs were visualized using a volcano plot (**Figure 1B**).

### Identification of Gene Co-Expression Modules

**Figure 2:**
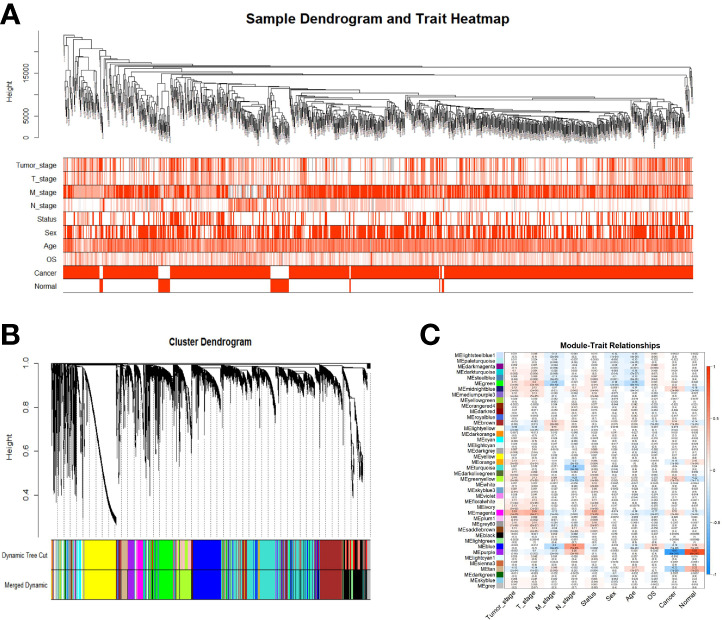
Identification of gene co-expression modules. **(A)** Expression Heat map of sample clustering and clinical features; **(B)** Area versus difference metric (1-TOM) dendrograms of gene expression quantities were clustered, and **(C)** Heat map of the correlation between module eigengenes and clinical features. Note: Color depth is positively correlated with clinical stage, TNM stage, age, and gray represents censored values. Red represents male, deceased samples, while white represents female, alive samples.

**Figure 3:**
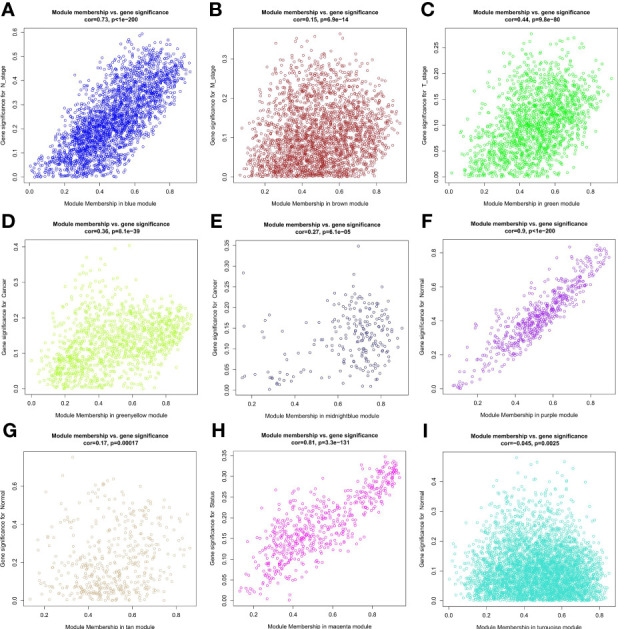
Correlation between module membership of the module in which the ferroptosis-associated genes are located and gene significance. **(A)** Blue module versus N stage; **(B)** Brown module versus M stage; **(C)** Green module versus T stage; **(D)** Green yellow module versus cancer; **(E)** Midbright blue versus cancer; **(F)** Purple module versus normal; **(G)** Tan module versus normal; **(H)** Magenta module versus status; **(I)** Turquoise module versus normal.

Samples were first clustered according to their Euclidean distance (**Figure 2A**), and the soft threshold was set to 4 (scale-free R^2^ = 0.83) to guarantee the construction of scale-free networks. A total of 42 modules were confirmed (**Figure 2B**), and the modules were correlated with clinical features (**Figure 2C**). As shown in **Table 1**, 60 ferroptosis-related genes were confirmed to be distributed in which gene module, respectively. The module membership in these modules was searched for highly correlated clinical features. The association with gene significance was confirmed to guarantee that genes highly significantly associated with this clinical feature were also significant elements in this module (**Figure 3**).

**Table 1:** 60-ferroptosis associated genes in gene modules.

Module	Gene
Blue	AKR1C3, CHAC1, CISD1, CS, GCLC, LPCAT3, TFRC, TP53, EMC2, ACSL3, NFE2L2, SLC1A5, GOT1
Brown	ALOX12
Green	DPP4, HMGCR, FDFT1, AIFM2, PEBP1, SQLE, FADS2, KEAP1
Green-yellow	ALOX5, CARS1, CD44, SAT1, FTH1, STEAP3, ABCC1, HMOX1
Grey	PTGS2
Magenta	FANCD2, SLC7A11
Midnight blue	NCOA4
Purple	ACSL4
Royal blue	ALOX15
Tan	MT1G, ACO1, ACSF2
Turquoise	AKR1C1, AKR1C2, ATP5MC3, GCLM, GLS2, GPX4, GSS, HSPB1, CRYAB, RPL8, PHKG2, HSBP1, NFS1, ACACA, ZEB1, NQO1, NOX1, G6PD, PGD, IREB2
Yellow	CBS

### Prognostic Analysis of Ferroptosis-Related Genes in Renal Cancer Patients

**Figure 4:**
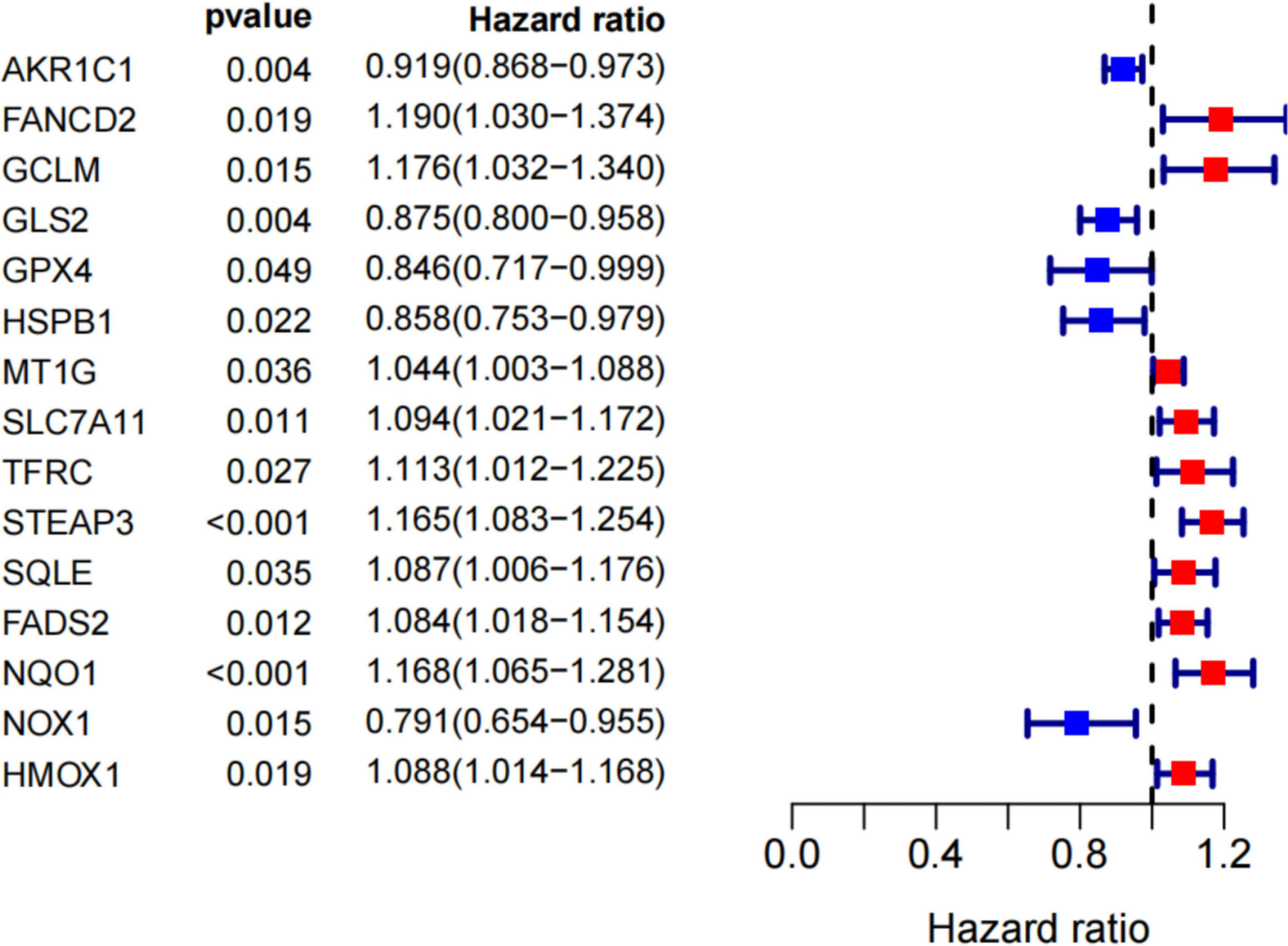
Search for genes associated with iron death in the prognosis of renal cancer patients by univariate COX analysis.

**Figure 5:**
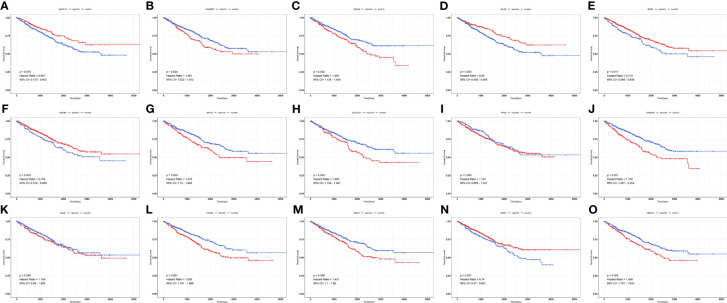
Survival curves of ferroptosis-associated genes in patients with renal cancer. **(A)** AKR1C1; **(B)** FANCD2; **(C)** GCLM; **(D)** GLS2; **(E)** GPX4; **(F)** HSPB1; **(G)** MT1G; **(H)** SLC7A11; **(I)** TFRC; **(J)** STEAP3; **(K)** SQLE; **(L)** FADS2; **(M)** NQO1; **(N)** NOX1; **(O)** HMOX1.

**Figure 6:**
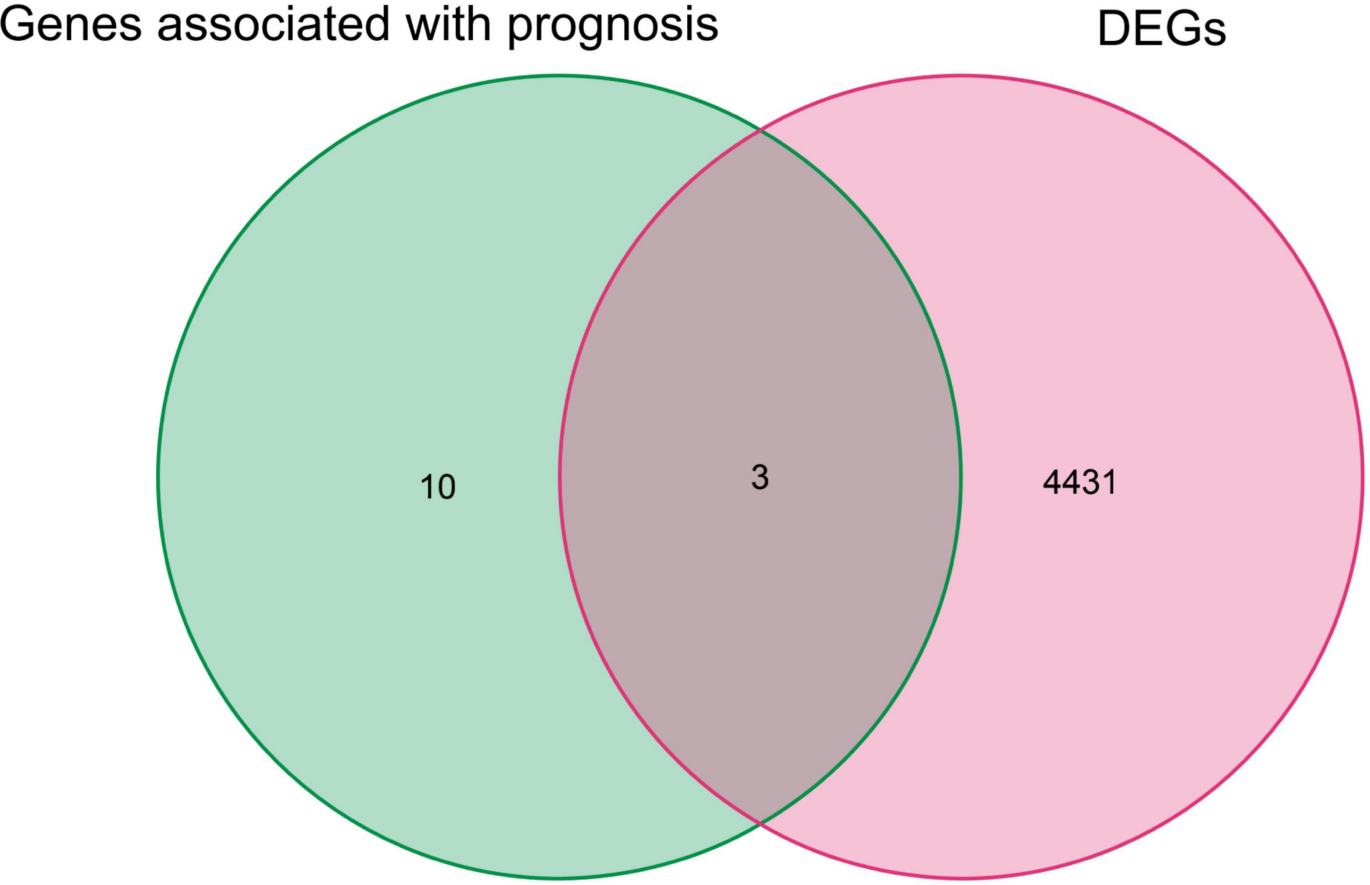
Genes overlapping between iron death genes and DEGs associated with prognosis in renal cancer patients.

By univariate Cox analysis, 15 ferroptosis-related genes (AKRIC1, FANCD2, GCLM, GLS2, GPX4, HSPB1, MT1G, SLC7A11, TFRC, STEAP3, SQLE, FADS2, NQO1, NOX1, and HMOX1) were found to be associated with renal cancer patient outcomes, as detailed in **Figure 4**. The results of the Kaplan–Meier test showed that the levels of TFRC and SQLE expression were not related to the survival of patients with renal cancer (**Figure 5**). The remaining 13 genes intersected with the DEGs to obtain three genes: SLC7A11, HMOX1, and MT1G (**Figure 6**).

### SLC7A11, HMOX1, and MT1G Expression in Renal Cancer

**Figure 7:**
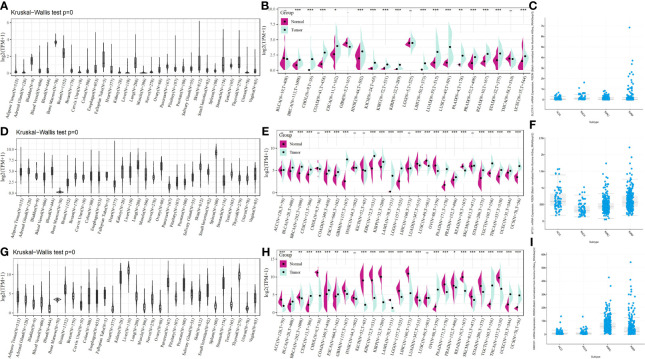
SLC7A11, HMOX1, and MT1G expression in pan cancer, as well as renal cancer subtypes. SLC7A11 expression in **(A)** different tissues; **(B)** different tumor tissues and normal tissues; **(C)** different renal cancer subtypes; HMOX1 expression in **(D)** different tissues; **(E)** different tumor tissues and normal tissues; **(F)** different renal cancer subtypes; MT1G expression in **(G)** different tissues; **(H)** different tumor tissues and normal tissues; **(I)** different renal cancer subtypes. * indicates P < 0.05; ** indicates P < 0.01; *** indicates P < 0.001. ACC: adrenocortical carcinoma; KICH: kidney chromophobe carcinoma; KIRC: kidney clear cell carcinoma; KIRP: kidney papillary cell carcinoma.

To provide a more comprehensive picture of SLC7A11, HMOX1, and MT1G expression, we used the Kruskal–Wallis test for the expression of these three genes in the GTEx database. SLC7A11 was found to be expressed at a low level in the kidney (**Figure 7A**), HMOX1 at a significantly lower level in the bone marrow (**Figure 7D**) and a medium level in the kidney, and MT1G at the highest levels in the kidney, liver, and thyroid (**Figure 7G**). The pan-cancer expression profiles of SLC7A11, HMOX1, and MT1G were subsequently analyzed by integrating tumor samples in TCGA with normal samples in GTEx using the rank-sum test. SLC7A11 was found to be expressed at high levels in tumor samples from KICH, KIRC, and KIRP, with upregulation of HMOX1 occurring in KIRC and KIRP. At the same time, MT1G was significantly downregulated in the four tumors (**Figures 7B, E, H**). SLC7A11, HMOX1, and MT1G expression in ACC, KICH, KIRC, and KIRP was mapped by the online tool called cBioPortal (**Figures 7C, F, I**).

### Prognostic Analysis of SLC7A11, HMOX1, MT1G in Patients With Different Renal Cancer

**Figure 8:**
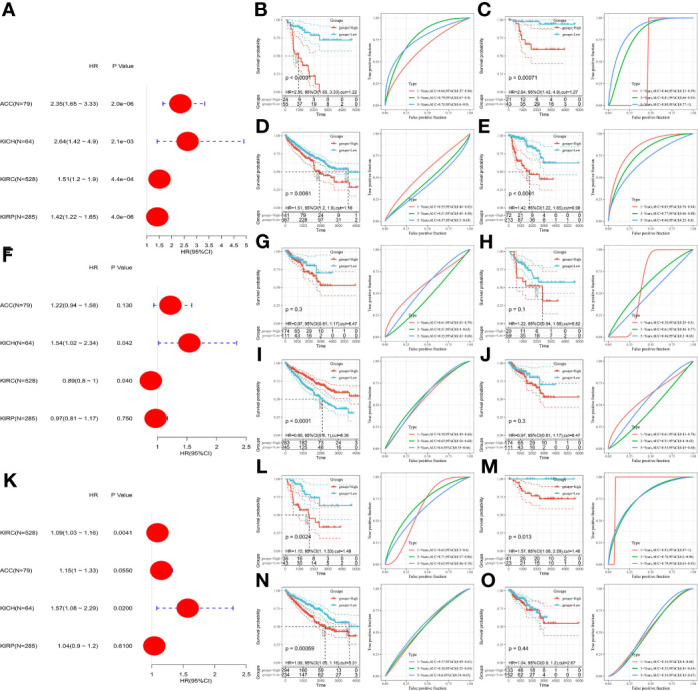
Prognostic analysis of SLC7A11, HMOX1, MT1G in patients with different renal cancer subtypes. **(A)** The association between SLC7A11 and prognosis in four renal cancer subtypes was assessed using univariate COX regression analysis; **(B–E)** the prognostic value of SLC7A11 in ACC, KICH, KIRC and KIRP; **(F)** The association between HMOX1 and prognosis in four renal cancer subtypes was assessed using univariate COX regression analysis; **(G–J)** the prognostic value of HMOX1 in ACC, KICH, KIRC and KIRP; **(K)** The association between MT1G and prognosis in four renal cancer subtypes was assessed using univariate COX regression analysis; **(L–O)** the prognostic value of SLC7A11 in ACC, KICH, KIRC and KIRP. ACC, adrenocortical carcinoma; KICH, kidney chromophobe carcinoma; KIRC, kidney clear cell carcinoma; KIRP, kidney papillary cell carcinoma.

Due to the high heterogeneity among the different renal cancer subtypes, as shown in **Figure 8**, the prognostic value of these three genes in the four renal cancer subtypes was evaluated by univariate Cox regression analysis and corrected using the Kaplan–Meier method and ROC curves. We found that SLC7A11 increased its risk of poor prognosis in four renal cancers (**Figures 8A–E**). High HMOX1 expression, although increasing the prognostic risk of KICH, Kaplan-Meier, and ROC results, indicated that it could not predict prognosis. In contrast, KIRC patients with low HMOX1 expression had a significantly increased proportion of poor prognosis outcomes, as detailed in **Figures 8F–J**. Interestingly, we found that high MT1G expression was a poor prognostic factor in ACC, KICH, KIRC and was not associated with prognostic survival in KIRP patients.

### Functional Annotation of SLC7A11, HMOX1, and MT1G

**Figure 9:**
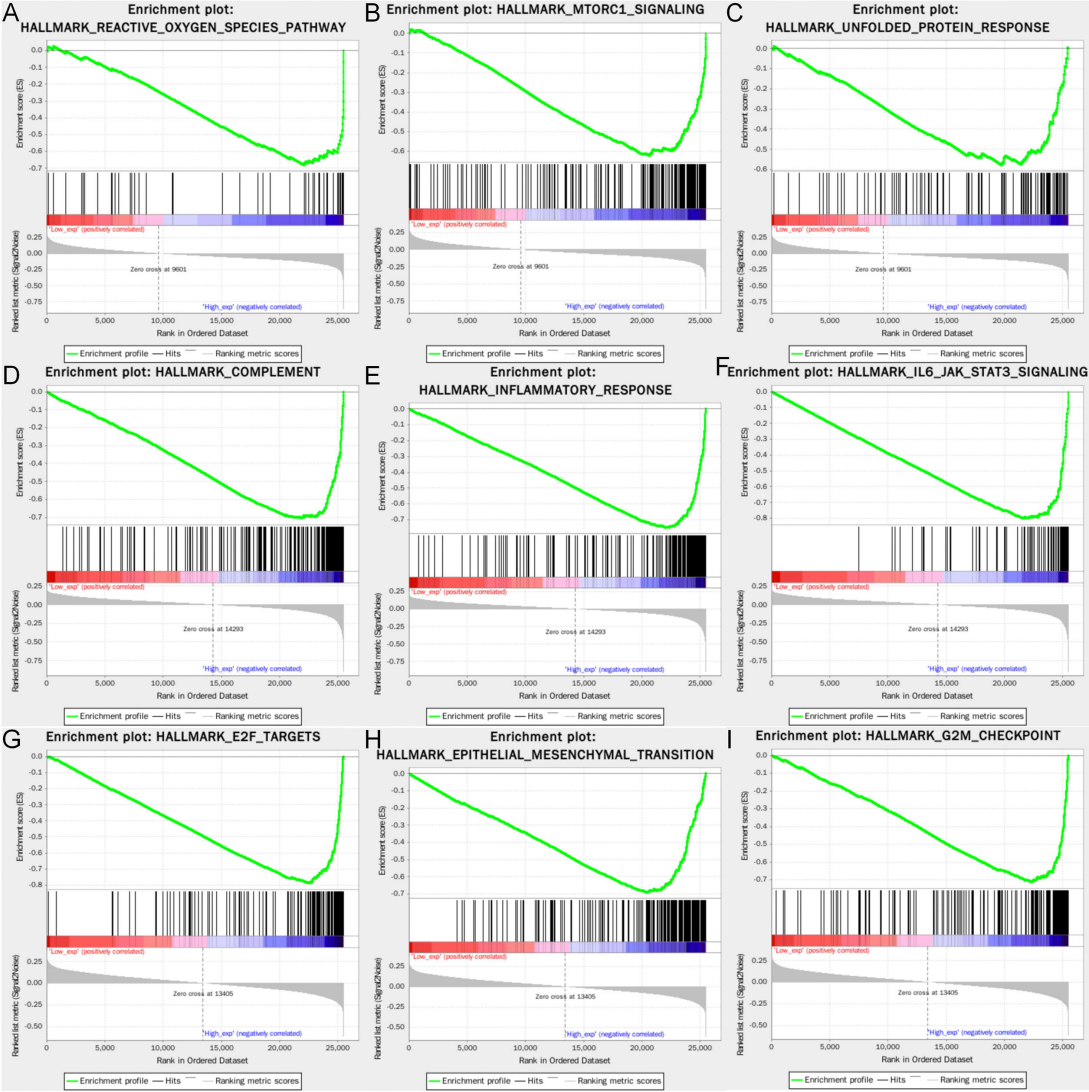
Gene set enrichment analysis of the three genes. The GSEA of **(A–C)** SLC7A11; **(D–F)** HMOX1; **(G–I)** MT1G. GSEA, Gene set enrichment analysis.

The three hallmark pathways most significantly associated with high expression of SLC7A11, HMOX1, and MT1G are presented in **Figure 9**. Among them, SLC7A11 overexpression was positive in terms of reactive oxygen species pathway, mTORC1 signaling, and unfolded protein response; HMOX1 overexpression was associated with complement, inflammatory response, and IL-6-JAK-STAT3 signaling pathway; while MT1G overexpression was also significantly enriched in E2F1 targets, epithelial-mesenchymal transition, and G2M checkpoint.

## Discussion

Ferroptosis is a cell death modality mainly driven by lipid peroxidation (17). When cellular ROS accumulate over the amount of redox required for GSH and phospholipid hydroperoxides, redox homeostasis is disrupted, triggering ferroptosis cell death. Alternatively, GPX4 can protect cells from ferroptosis by scavenging lipid peroxides in the cell, utilizing GSH. Therefore, deletion or inhibition of glutathione peroxidase 4 (GPX4) triggers ferroptosis even when GSH and cysteine contents in the cell are normal (17, 18). It is currently found that most cancers have higher levels of ferroptosis in their tumor tissues compared with adjacent normal tissues, are associated with drug sensitivity, cancer metastasis, clinical features, and clinical outcomes; it is also found that ferroptosis-related genes are differentially expressed in cancers and that different genes exhibit different modes of regulation in various cancers, with a high degree of tumor specificity (19). Inducing or preventing tumor cell ferroptosis by targeting ferroptosis-related genes is currently a promising therapeutic approach in cancer treatment.

Recent studies have demonstrated that iron-mediated ferroptosis is a crucial factor in the pathogenesis of acute kidney injury and acute renal failure, and iron homeostasis can serve as a therapeutic target for acute kidney injury, effectively preventing or attenuating tissue damage in the kidney by upregulating GPX4 (20–22). Targeting ferroptosis-related genes such as GPX4, AIFM2, and HDAC can modulate oxidative stress and thus confer susceptibility to ferroptosis in tumor cells (23, 24). Recently, Wu and Li et al. performed the construction of a prognostic prediction model for ferroptosis-related genes for KIRC (25, 26). Upregulation of ferroptosis-related gene expression is positively correlated with KIRC disease progression, and survival models based on ferroptosis-related genes may provide a promising predictor of prognosis for KIRC patients. Based on the tissue specificity of iron homeostasis for the kidney and the study of pro-ferroptosis proteins in cancer prognosis, it is reasonable to speculate that there is great value yet to be tapped in the prognostic evaluation of the pan-renal cancer.

We identified three gene signatures associated with renal cancer prognosis in this study. Still, we seem to observe some interesting phenomena. After splitting the different renal cancer subtypes, the prognostic value of these gene signatures was altered compared to that of the overall renal cancer samples before. According to the WGCNA results, the blue module—where SLC7A11 is located—is significantly and positively associated with patient survival status; the green and yellow modules—where SLC7A11 is located—are associated with cancer; and the tan module, where MT1G is located, is weaklier related to normal. Survival analysis was performed on the whole renal cancer samples in TCGA, and high expression of SLC7A11, HMOX1 and MT1G all presented some prognostic risk. Patients with increased expression of these genes showed significantly decreased survival. After the different types of kidney cancer were disassembled for analysis, high levels of SLC7A11 remained significantly associated with poor prognosis in patients with ACC, KICH, KIRC, and KIRP, suggesting that SLC7A11 could be used as a potential biological marker for the prognostic evaluation of patients with various types of kidney cancer.

SLC7A11 is a multipass transmembrane protein that mediates cystine–glutamate antiporter activity in system X (27). In many cancer cells, SLC7A11 is adaptively upregulated to alleviate the stress imposed by intracellular ROS and promote GSH synthesis and resistance to ferroptosis, promote tumor growth by inhibiting ferroptosis, and resist resistance to anticancer therapy (27–29). Currently, there are small molecule inhibitors targeting SLC7A11, such as sorafenib, sulfasalazine, etc., which can play a therapeutic role in many cancers by inhibiting SLC7A11 activity (28, 30). SLC16A1 overexpression was also found in Wu’s study to be an independent risk factor associated with KIRC prognosis (25). Our study extends on this basis that SLC7A11 has favorable prognostic ability in other types of renal cancer as well.

Nevertheless, we found that the high level of expression of HMOX1 was no longer a poor prognostic factor in various subtypes of renal cancer, and its assessment for the poor prognosis of KICH did not seem to be more accurate. Conversely, low HMOX1 expression paradoxically caused decreased survival in KIRC patients. We, therefore, speculate that high HMOX1 expression does not serve as a poor prognostic factor for overall renal cancer patients due to the high heterogeneity among different subtypes of renal cancer. HMOX1 is a cytoprotective enzyme in response to cellular stress. It exhibits anti-apoptotic, anti-inflammatory, and anti-ferroptosis properties, and HOMX1 attenuates renal proximal tubule cell ferroptosis triggered by adrenocortical hormones (31). However, when iron ions and ROS content in cells become overloaded, HMOX1 is excessively activated, and HOMX1 is converted from a protector to a detriment to induce cell ferroptosis (32). Lin (33) and others also showed in their study that EF24, a synthetic analogue of curcumin, was found to upregulate HMOX1 by increasing MDA, ROS, and ferric ion levels in cells, which in turn inhibited GPX4 activity and induced ferroptosis in osteosarcoma cells, which also flanked the results of our study and suggested that low HMOX1 expression may be a monitoring marker of poor prognosis in KIRC patients.

In addition to this, patients with high MT1G expression also exhibited poor prognostic outcomes in ACC, KICH, and KIRP, which may be related to the unique pathogenesis of KIRP, which is independent of disease progression and clinical outcomes in addition to KIRC, despite its exact origin (34). MT1G belongs to the metallothionein superfamily and can be highly inducible in response to stress factors, including metal ions (35), and MT1G has been reported to inhibit lipid peroxidation mediated ferroptosis, protect cells from sorafenib injury and promote tumor growth in hepatocellular carcinoma. Therefore, MT1G has also emerged as a critical target to overcome acquired resistance to sorafenib (36). Besides, Lin et al. also successfully constructed a prognostic model including MT1G and confirmed that high MT1G expression was associated with low OS in KIRC patients (37). The GSEA also revealed that high MT1G expression was related to pro-oncogenic signaling pathways such as E2F1 targets, epithelial-mesenchymal transition, and G2M checkpoint, further suggesting that MT1G may act as a relevant gene to suppress ferroptosis and adversely affect the prognosis of renal cancer patients.

In summary, we systematically addressed the prognostic ability of SLC7A11, HMOX1, and MT1G in overall renal cancer and different renal cancer subtypes in this study. However, considering the limitations of the experimental conditions, we did not have more opportunities to deeply explore the specific mechanisms of these genes in renal cancer-associated ferroptosis; as a result, how SLC7A11, HMOX1, and MT1G induce ferroptosis in different subtypes of renal cancer cells remains unknown; nevertheless, it will be the main direction of our subsequent studies. In summary, the present study provides a biological marker associated with ferroptosis for the prognosis of renal cancer patients that may help clinical decision-making for individualized treatment of renal cancer.

## Data Availability Statement

The original contributions presented in the study are included in the article/supplementary material. Further inquiries can be directed to the corresponding authors.

## Author Contributions

XC and GZ designed the analysis flow. LC and CW performed the analysis. LC, YW, and TH participated in writing the manuscript. All authors have read and participated in the revision of the manuscript.

## Conflict of Interest

The authors declare that the research was conducted in the absence of any commercial or financial relationships that could be construed as a potential conflict of interest.

## Publisher’s Note

All claims expressed in this article are solely those of the authors and do not necessarily represent those of their affiliated organizations, or those of the publisher, the editors and the reviewers. Any product that may be evaluated in this article, or claim that may be made by its manufacturer, is not guaranteed or endorsed by the publisher.
